# Prevalence of Suicidal Behavior and Self-Injury Endorsement Among Saudi Medical Students: A Cross-Sectional Study of Academic Year Trajectories and Associated Factors

**DOI:** 10.3390/healthcare14101295

**Published:** 2026-05-11

**Authors:** Bander A. Haddad

**Affiliations:** Psychiatry Department, Imam Mohammad Ibn Saud Islamic University (IMSIU), Riyadh 11623, Saudi Arabia; bahaddad@imamu.edu.sa

**Keywords:** suicidal behavior, self-injury, medical students, self-esteem, Saudi Arabia, mental health

## Abstract

Objective: This study examined the prevalence and correlates of suicidal behavior and self-injury endorsement among Saudi medical students, with particular attention to academic year trajectories. Method: A cross-sectional survey was conducted among 1138 medical students from colleges across all five Saudi administrative regions. Outcomes were self-reported endorsement of suicidal behaviors and self-injury behaviors. The Rosenberg Self-Esteem Scale assessed self-esteem. Multivariable logistic regression identified independently associated factors. Results: The prevalence of suicidal behavior endorsement was 17.0% (95% CI: 14.9–19.3) and self-injury endorsement was 16.9% (95% CI: 14.8–19.2). Both outcomes peaked during the 2nd year of training (suicidal: 24.1%; self-injury: 26.3%) and were significantly higher among pre-clinical compared to clinical-stage students (suicidal: 21.5% vs. 13.8%, *p* < 0.001; self-injury: 20.9% vs. 14.1%, *p* = 0.003). In multivariable analysis, pre-clinical stage (AOR = 1.69, *p* = 0.019), living alone (AOR = 1.81, *p* = 0.025), current smoking (AOR = 2.27, *p* < 0.001), chronic disease (AOR = 2.19, *p* = 0.001), and diagnosed mental illness (AOR = 2.35, *p* < 0.001) were independently associated with suicidal behavior endorsement. High self-esteem was strongly associated with lower odds of both outcomes (suicidal: AOR = 0.19, *p* = 0.003; self-injury: AOR = 0.32, *p* = 0.014). Conclusions: Saudi medical students demonstrate substantial prevalence of suicidal behavior and self-injury endorsement, with a pronounced vulnerability window during the pre-clinical years. Findings support targeted mental health screening and intervention during early medical training, with self-esteem enhancement as a potentially modifiable protective factor.

## 1. Introduction

Medical students constitute a population at elevated risk for psychological distress due to sustained academic pressure, high workload, sleep disruption, and repeated exposure to human suffering [1,2]. Systematic reviews and meta-analyses consistently report high rates of depression, anxiety, and burnout during medical training, alongside clinically important levels of suicidal ideation [3,4,5]. These burdens may substantially exceed those observed in age-matched peers and the general population.

Suicidal ideation and self-injury are two critical but distinct manifestations of self-harm-related psychopathology. Suicidal ideation encompasses thoughts of ending one’s life, whereas self-injury involves deliberate self-inflicted harm and may occur with or without suicidal intent. Non-suicidal self-injury (NSSI) refers specifically to self-injury without conscious suicidal intent and is prevalent in adolescents and young adults [6,7]. Importantly, self-injury is strongly associated with subsequent suicide attempts and death by suicide [8,9].

The temporal dynamics of psychological distress across medical training have emerged as an area of increasing scholarly inquiry. Evidence suggests that distress may not distribute uniformly across training years, with several investigations identifying the pre-clinical years as a period of heightened vulnerability [10,11]. The transition from undergraduate education to the demanding medical curriculum, coupled with the intensity of foundational science coursework and high-stakes examinations, may render early-year students particularly susceptible to psychological difficulties [12]. Conversely, progression to clinical years, while introducing new stressors related to patient care responsibilities, may also confer protective factors including clearer professional identity formation, clinical engagement providing meaning and purpose, and accumulated coping strategies [13,14]. However, the literature examining academic year trajectories specifically for suicidal ideation and self-injury remains limited, with most studies reporting aggregate prevalence without stratification by training stage.

The Middle Eastern and North African (MENA) region presents a distinctive sociocultural context in which collectivist family structures, religious norms, and stigma surrounding mental illness may influence disclosure and help-seeking [15,16,17,18]. Saudi Arabia has also experienced rapid expansion in medical education; for example, the number of medical colleges increased from 5 to 21 between 2001 and 2009 [19]. Despite this growth, region-specific epidemiological data on suicidal behavior and self-injury among Saudi medical students remain limited [20].

Saudi Arabia’s medical education system follows a six-year undergraduate model. The first three years (pre-clinical phase) focus on foundational biomedical sciences delivered through intensive didactic instruction and high-stakes examinations. The subsequent three years (clinical phase), including a final internship year, involve direct patient care responsibilities, clinical rotations across specialties, and progressive clinical decision-making. The transition from pre-clinical to clinical training represents a fundamental shift in learning demands, from memorization-heavy theoretical coursework to applied clinical reasoning and patient interaction. This structure provides a natural framework for examining whether psychological vulnerability varies across distinct training phases.

Epidemiological data on suicidal behavior in Saudi Arabia remain limited, though available evidence suggests a non-trivial burden. The Saudi National Mental Health Survey [21], a nationally representative household survey, provided the first population-level estimates of suicidal thoughts and behaviors in the Kingdom. Among Saudi medical students specifically, Madadin et al. [22] reported a suicidal ideation prevalence of 8.6% among medical students in Dammam. Data on non-suicidal self-injury in Saudi populations are particularly scarce; a recent systematic review by Bahamdan and Aldhawyan [23] synthesized the limited available evidence on NSSI in the Saudi general population but noted significant gaps, particularly among university students.

Self-esteem, conceptualized as an individual’s global evaluation of self-worth, is a psychologically salient construct during emerging adulthood [24]. Prospective evidence indicates that low self-esteem predicts subsequent depressive symptoms and broader psychological distress [25]. Within medical training, self-esteem may shape vulnerability to academic and clinical stressors, coping responses, and help-seeking behavior, and therefore may be relevant to self-injury and suicidal outcomes.

Additional factors implicated in medical student suicidal behavior include smoking and substance use, which may serve as maladaptive coping mechanisms or indicators of broader psychological distress [26,27]. Chronic medical conditions have similarly been associated with elevated depression and suicidal ideation risk, potentially mediated through functional limitations, pain, or disease-specific psychological impacts [28]. Mental illness diagnosis represents perhaps the most robust predictor, yet stigma and help-seeking barriers may result in delayed recognition and treatment within medical student populations [29,30].

Despite the growing body of literature on medical student mental health, relatively fewer studies have examined suicidal behavior and self-injury in relation to self-esteem, particularly within Saudi Arabian medical education. To our knowledge, year-by-year patterns of suicidal behavior and self-injury endorsement across the medical curriculum—and their association with self-esteem—have not been well characterized in large multicenter samples from Saudi Arabia.

The present study addresses these gaps through a comprehensive cross-sectional examination of suicidal behavior and self-injury endorsement among medical students across Saudi Arabia. Our primary objectives were: (1) to determine the prevalence of suicidal behavior and self-injury endorsement among Saudi medical students; (2) to examine whether endorsement prevalence varies across academic training stages, specifically comparing pre-clinical and clinical years; and (3) to identify demographic, behavioral, and psychological factors independently associated with endorsement. We hypothesized that pre-clinical students would demonstrate elevated endorsement rates compared to their clinical-year counterparts, and that diagnosed mental illness, smoking, chronic disease, and low self-esteem would emerge as significant independent predictors.

## 2. Method

### 2.1. Study Design and Setting

This cross-sectional study employed secondary analysis of data originally collected to examine self-esteem and associated behaviors among medical students in Saudi Arabia [31]. The primary study was conducted between March 2022 and December 2022, recruiting participants from medical colleges across all five administrative regions of Saudi Arabia: Central, Northern, Southern, Eastern, and Western. Both government and private medical institutions were represented. The present secondary analysis examines suicidal behavior and self-injury endorsement as primary outcomes, with particular attention to academic year trajectories—variables that were collected but not comprehensively analyzed in the parent study.

Saudi Arabia’s medical education system follows a six-year undergraduate model comprising three pre-clinical years focused on foundational biomedical sciences followed by three clinical years including a final internship year. This structure provided a natural framework for examining psychological outcomes across distinct training phases.

### 2.2. Participants and Sampling

The original study employed convenience sampling through electronic distribution of questionnaires via social media platforms. Eligibility criteria included current enrollment in a medical program at any Saudi Arabian medical college, regardless of nationality, gender, or academic year. The sole exclusion criterion was non-medical student status.

Sample size estimation for the original study assumed 50% response distribution, 95% confidence level, and 5% margin of error, yielding a minimum required sample of 379 participants. The achieved sample substantially exceeded this threshold. Of 1188 initial responses, 50 respondents indicated they were not medical students and were excluded, yielding an analytic sample of 1138 participants for the present secondary analysis. Medical colleges were invited through existing academic networks, and participating colleges distributed the survey link to all enrolled students via institutional email and learning platforms. Participation was voluntary and anonymous, and no incentives were provided. The decentralized distribution model, while enabling broad geographic coverage, meant that participating colleges did not systematically track the number of students who received the survey link, precluding calculation of institution-level or aggregate response rates. No sampling weights were applied, and all analyses treated the sample as an unweighted convenience sample. Partially completed questionnaires with missing outcome data were excluded from analysis; remaining missing values on covariates were handled by listwise deletion in the regression models.

The gender distribution in the sample (55.5% female) is broadly consistent with national trends showing increasing female enrollment in Saudi medical colleges, though precise national-level gender ratios for medical students are not centrally reported. The regional distribution reflects the geographic concentration of medical colleges, with the Central region (housing multiple universities in Riyadh) most heavily represented.

### 2.3. Ethical Considerations

The original study received ethical approval from the Institutional Review Board of the Medical Research Unit, Faculty of Medicine, Imam Mohammad Ibn Saud Islamic University (IMSIU), Riyadh, Saudi Arabia (Research Project No. 201/2022). All procedures adhered to the Declaration of Helsinki. Informed consent was obtained electronically from all participants prior to questionnaire completion. Participation was voluntary, anonymous, and uncompensated. The survey introduction provided information about available university counseling services and national crisis helplines, and all participants were encouraged to seek help if they experienced distress while completing the questionnaire. Contact details for on-campus mental health services and 24 h telephone support lines were displayed on the final page of the survey. No direct real-time risk assessment or active follow-up was possible because the survey was anonymous and no identifying information was collected.

## 3. Measures

### 3.1. Primary Outcomes

The primary outcomes were endorsement of suicidal behavior and endorsement of self-injury behavior, assessed using two single items from the behavioral questionnaire developed for the parent study. Participants responded to the statements “Suicidal behaviors” and “Self-injury behaviors” on a four-point Likert scale (Strongly disagree, Disagree, Agree, Strongly agree). Responses were dichotomized into endorsement (Agree or Strongly agree) versus non-endorsement (Disagree or Strongly disagree). Two secondary composite outcomes were also examined: “any endorsement,” defined as endorsing either suicidal behavior or self-injury behavior (or both), and “co-occurring endorsement,” defined as endorsing both behaviors. These self-report items did not differentiate non-suicidal self-injury from suicidal self-harm in terms of intent, nor did they capture frequency, recency, or method of the behaviors.

### 3.2. Primary Exposure

The primary exposure variable was academic training stage, operationalized as a binary variable distinguishing pre-clinical students (1st, 2nd, and 3rd year) from clinical-stage students (4th year, 5th year, and Intern). Secondary analyses examined the six individual academic year categories to characterize prevalence trajectories with greater granularity.

#### 3.2.1. Self-Esteem Assessment

Self-esteem was measured using the 10-item Rosenberg Self-Esteem Scale (RSES) [32]. Items are rated on a 4-point Likert scale (0 = strongly disagree to 3 = strongly agree). Negatively worded items are reverse-scored, yielding a total score ranging from 0 to 30, with higher scores indicating higher self-esteem. Consistent with the parent study, scores were categorized as low (<15), normal (15–25), and high (>25). Internal consistency in the present sample was good (Cronbach’s α = 0.83).

#### 3.2.2. Covariates

Sociodemographic variables included age (categorized as 18–20, 21–23, 24–26, or >26 years), gender (male or female), and geographic region of medical college (Central, Northern, Southern, Eastern, or Western). Academic performance was assessed through self-reported cumulative grade point average (GPA) on the Saudi 5.0 scale, categorized as 4.75–5.00, 4.50–4.74, 4.00–4.49, 3.00–3.99, or <3.00. Living arrangement was classified as living with family, living alone, or living with a colleague. Health-related variables included smoking status (current smoker vs. non-smoker, encompassing cigarettes, electronic cigarettes/vaping, and shisha), presence of chronic disease (yes/no), and history of diagnosed mental illness (yes/no). Participants endorsing mental illness diagnosis were asked to specify the condition.

### 3.3. Statistical Analysis

All analyses were conducted using IBM SPSS Statistics for Windows, version 26.0 (IBM Corp., Armonk, NY, USA). Statistical significance was defined as two-tailed *p* < 0.05.

Sample characteristics were summarized using frequencies and percentages for categorical variables. Continuous variables were described using means and standard deviations. Outcome prevalence was reported as percentages with 95% confidence intervals.

Descriptive statistics were computed for all study variables. Prevalence estimates are reported as proportions with 95% confidence intervals (CIs) calculated using the Wilson method. Differences in endorsement across academic years and between pre-clinical versus clinical/intern stages were evaluated using Pearson’s chi-square (χ^2^) tests. A two-sided *p* value < 0.05 was considered statistically significant.

Multivariable logistic regression models were constructed to estimate adjusted odds ratios (AORs) and 95% CIs for suicidal behavior endorsement and self-injury endorsement, adjusting for potential confounders. The adjustment set included training stage, gender, age group, region, GPA, living status, smoking, chronic disease, diagnosed mental illness, and self-esteem category. Model discrimination was assessed using the area under the receiver operating characteristic curve (AUC), and overall model fit was summarized using likelihood ratio χ^2^ statistics and McFadden’s pseudo-R^2^. Analyses were conducted in IBM SPSS (Version 26.0).

## 4. Results

### 4.1. Participant Characteristics

A total of 1138 medical students were included in the analysis following exclusion of 50 non-medical student respondents from the initial 1188 responses. Table 1 presents the sociodemographic and clinical characteristics of the sample. The mean age was 22.4 years, with the majority (50.4%) falling within the 21–23 age range. Female students comprised 55.5% of the sample (n = 632). Participants represented all six academic years, with the largest proportions from 5th year (23.6%), 4th year (19.9%), and 3rd year (19.1%). When dichotomized by training stage, 470 students (41.3%) were in pre-clinical years (1st–3rd) and 668 (58.7%) were in clinical years or internship.

Participants were distributed across all five Saudi administrative regions, with the Central region most heavily represented (33.2%), followed by Northern (21.6%) and Southern (20.5%) regions. The majority of students (84.4%) lived with their families. Regarding academic performance, 41.8% reported GPAs of 4.50 or higher on the 5.0 scale.

One-fifth of participants (20.7%) reported current smoking. Chronic disease was reported by 12.4% of the sample, while 18.2% reported having been diagnosed with a mental illness. Among the 207 participants reporting a diagnosed mental illness, the most commonly reported diagnoses were anxiety disorders (n = 116, 56.0%), depression (n = 103, 49.8%), psychotic disorders (n = 23, 11.1%), bipolar disorder (n = 22, 10.6%), and phobia (n = 15, 7.2%). Less frequently reported conditions included obsessive–compulsive disorder (n = 5, 2.4%), attention-deficit/hyperactivity disorder (n = 4, 1.9%), borderline personality disorder (n = 7, 3.4%), post-traumatic stress disorder (n = 3, 1.4%), and panic disorder (n = 3, 1.4%). Multiple diagnoses were permitted, reflecting comorbidity within this subgroup. The mean Rosenberg Self-Esteem Scale score was 17.91 (SD = 5.36). When categorized, 23.9% of participants had low self-esteem, 67.6% had normal self-esteem, and 8.5% had high self-esteem.

### 4.2. Prevalence of Suicidal Behavior and Self-Injury Endorsement

Overall prevalence rates are presented in Table 2. Among the 1138 participants, 192 (16.9%; 95% CI: 14.8–19.2) endorsed self-injury behaviors and 193 (17.0%; 95% CI: 14.9–19.3) endorsed suicidal behaviors. When examining composite outcomes, 259 participants (22.8%; 95% CI: 20.4–25.3) endorsed at least one of the two behaviors, while 126 (11.1%; 95% CI: 9.4–13.0) endorsed both behaviors concurrently.

### 4.3. Academic Year Trajectories

**Figure 1:**
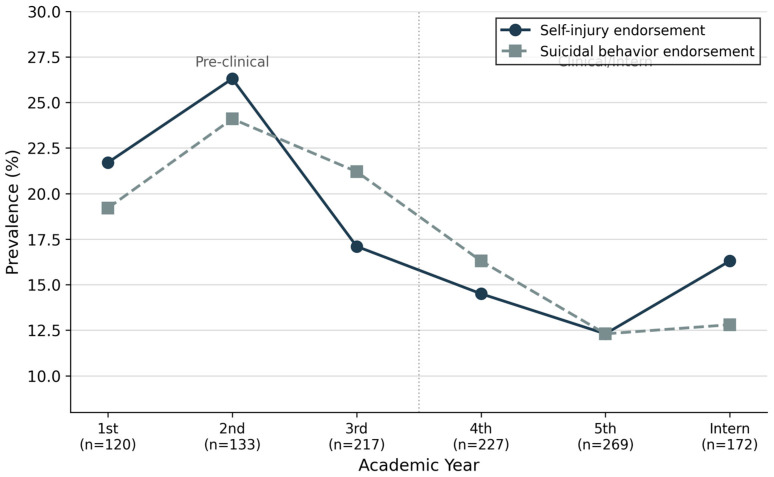
Note. Chi-square tests: Self-injury endorsement χ^2^(5) = 15.42, *p* = 0.009; Suicidal behavior endorsement χ^2^(5) = 14.34, *p* = 0.014. *Prevalence of Suicidal Behavior and Self-Injury Endorsement Across Academic Years. Note.* The vertical dotted line demarcates pre-clinical (1st–3rd year) and clinical/intern (4th year–Intern) training stages. Sample sizes are indicated for each academic year.

Table 3 and Figure 1 present the prevalence of suicidal behavior and self-injury endorsement stratified by academic year. Both outcomes demonstrated a consistent pattern: prevalence was highest during the 2nd year of medical training (suicidal behavior: 24.1%; self-injury: 26.3%), followed by a progressive decline through subsequent years, with the lowest rates observed among 5th-year students (12.3% for both outcomes). A modest re-elevation in self-injury endorsement was observed during the intern year (16.3%). Chi-square tests confirmed statistically significant variation across academic years for both self-injury (χ^2^(5) = 17.94, *p* = 0.003) and suicidal behavior (χ^2^(5) = 14.89, *p* = 0.011). When dichotomized by training stage, pre-clinical students demonstrated significantly higher endorsement rates compared to clinical/intern students for both suicidal behavior (21.5% vs. 13.8%, *p* < 0.001) and self-injury (20.9% vs. 14.1%, *p* = 0.003).

### 4.4. Factors Associated with Suicidal Behavior Endorsement

Results of the multivariable logistic regression model for suicidal behavior endorsement are presented in Table 4. The model demonstrated acceptable discrimination (AUC = 0.754) and fit (McFadden pseudo-R^2^ = 0.143).

After adjustment for all covariates, pre-clinical training stage remained significantly associated with increased odds of suicidal behavior endorsement (AOR = 1.693, 95% CI: 1.090–2.631, *p* = 0.019). Students living alone had significantly elevated odds compared to those living with family (AOR = 1.808, 95% CI: 1.077–3.037, *p* = 0.025). Regional differences emerged, with students in the Northern region demonstrating higher odds than those in the Central region (AOR = 1.717, 95% CI: 1.075–2.742, *p* = 0.024).

Self-esteem demonstrated a strong protective gradient. Compared to students with low self-esteem, those with normal self-esteem had 36% lower odds of endorsement (AOR = 0.641, 95% CI: 0.443–0.928, *p* = 0.018), while those with high self-esteem had 81% lower odds (AOR = 0.192, 95% CI: 0.066–0.560, *p* = 0.003).

Three health-related factors were independently associated with increased odds: current smoking (AOR = 2.270, 95% CI: 1.514–3.404, *p* < 0.001), chronic disease (AOR = 2.192, 95% CI: 1.406–3.419, *p* = 0.001), and diagnosed mental illness (AOR = 2.353, 95% CI: 1.566–3.534, *p* < 0.001). Gender, age, and GPA were not significantly associated with suicidal behavior endorsement in the adjusted model.

### 4.5. Factors Associated with Self-Injury Endorsement

Results for self-injury endorsement are presented in Table 5. This model demonstrated acceptable discrimination (AUC = 0.708) and fit (McFadden pseudo-R^2^ = 0.102).

In contrast to the suicidal behavior model, pre-clinical training stage was not significantly associated with self-injury endorsement after adjustment (AOR = 1.236, 95% CI: 0.792–1.928, *p* = 0.350). However, younger age emerged as a significant predictor: students aged 18–20 had more than twice the odds of endorsement compared to those aged 21–23 (AOR = 2.124, 95% CI: 1.316–3.427, *p* = 0.002).

High self-esteem remained protective against self-injury endorsement (AOR = 0.319, 95% CI: 0.129–0.790, *p* = 0.014), though the protective effect of normal self-esteem did not reach statistical significance in this model (AOR = 0.801, 95% CI: 0.551–1.165, *p* = 0.246).

Current smoking (AOR = 2.453, 95% CI: 1.646–3.656, *p* < 0.001), chronic disease (AOR = 1.790, 95% CI: 1.145–2.799, *p* = 0.011), and diagnosed mental illness (AOR = 1.901, 95% CI: 1.263–2.861, *p* = 0.002) were all independently associated with increased odds of self-injury endorsement. Living arrangement approached but did not reach significance (living alone: AOR = 1.645, 95% CI: 0.981–2.759, *p* = 0.059). Gender, region, and GPA were not significant predictors.

## 5. Discussion

This study provides a multicenter examination of suicidal behavior and self-injury endorsement across academic training stages among Saudi medical students. Approximately one in six students endorsed suicidal behaviors (17.0%) and self-injury behaviors (16.9%), with prevalence peaking during the second year of medical training. Pre-clinical students demonstrated elevated endorsement rates compared to their clinical/intern counterparts, and multivariable analyses identified a set of potentially modifiable correlates that can inform targeted prevention and support strategies.

### 5.1. Prevalence in Context

The observed prevalence of suicidal behavior endorsement (17.0%) in our sample exceeds the 11.1% pooled estimate reported in the landmark meta-analysis by Rotenstein et al. [5], which synthesized data from 24 studies encompassing over 35,000 medical students globally. This discrepancy warrants careful interpretation. Methodological differences in outcome assessment likely contribute: our single-item endorsement measure captures a broader construct than instruments specifically designed to assess suicidal ideation with clinical precision. Nevertheless, the magnitude of endorsement observed—affecting nearly one-fifth of students—signals a substantial mental health burden within Saudi medical education that demands institutional attention.

Regional data remain comparatively limited. Nonetheless, available studies from neighboring countries suggest that medical trainees in the Gulf may experience substantial psychological morbidity. For example, Al-Maashani et al. [33] reported a high burden of depressive symptoms among medical students in Oman, and Amiri et al. [34] described lifetime suicidal behavior and attitudes among medical students in the United Arab Emirates. Together with our findings, these data underscore the need for culturally sensitive prevention and support strategies across the region.

The self-injury endorsement prevalence (16.9%) observed in our study is notable given the limited data on non-suicidal self-injury among medical students specifically. In the general young adult population, lifetime NSSI prevalence estimates range from 13% to 23% internationally [7]. The substantial overlap between self-injury and suicidal behavior endorsement in our sample—with 11.1% endorsing both—is consistent with theoretical models positing NSSI as both a risk factor for and correlate of suicidal ideation [8,35].

### 5.2. Academic Year Trajectories and the Pre-Clinical Vulnerability Window

Perhaps our most clinically significant finding is the identification of a pronounced vulnerability window during the early years of medical training, with 2nd-year students demonstrating peak endorsement for both suicidal behavior (24.1%) and self-injury (26.3%). This pattern—elevated risk during pre-clinical years followed by progressive decline through clinical training—has not been previously documented for these specific outcomes in the literature, though it aligns with broader observations regarding medical student distress trajectories.

Several mechanisms may underlie this temporal pattern. First, the transition into medical school represents a period of profound identity disruption and academic recalibration. Students who excelled in pre-medical education may encounter, often for the first time, the experience of relative academic struggles when competing against similarly high-achieving peers [13]. Second, the pre-clinical curriculum in Saudi medical schools emphasizes intensive foundational science coursework with high-stakes examinations, creating sustained stress without the meaning-making opportunities afforded by patient care [36]. Third, professional identity formation—a process increasingly recognized as protective for medical student wellbeing—remains nascent during pre-clinical years, leaving students without the psychological anchor of emerging physician identity [37].

The decline in endorsement rates observed from 3rd year onward may reflect multiple adaptive processes. Clinical immersion provides direct exposure to the purpose and meaning of medical training, potentially enhancing sense of coherence and resilience [4]. Students who experience severe psychological difficulties during early training may disproportionately withdraw from medical school, creating survivorship effects in later cohorts. Additionally, accumulated coping strategies, peer support networks, and help-seeking behaviors may develop over training duration, conferring progressive protection [38].

The modest elevation in self-injury endorsement during the intern year (16.3% vs. 12.3% in 5th year) deserves attention. Internship introduces distinct stressors including clinical responsibility, long working hours, sleep deprivation, and exposure to patient suffering and death. This pattern suggests that while the pre-clinical period represents a period of heightened vulnerability, the transition to independent clinical practice may introduce secondary risk that warrants monitoring.

### 5.3. The Protective Role of Self-Esteem

Our multivariable analyses identified several factors independently associated with endorsement that carry implications for screening and support. High self-esteem was strongly associated with reduced odds of suicidal behavior and self-injury endorsement. Although cross-sectional data cannot establish causality, self-esteem may index broader psychological resources (e.g., perceived self-efficacy, coping, and social connectedness) that reduce vulnerability to distress and self-harm-related outcomes [25,39]. Accordingly, wellness efforts that strengthen mentoring, coping skills, and access to timely mental health care may be particularly important for students with low self-esteem. Notably, attempts to increase self-esteem in isolation have shown mixed evidence for broader downstream benefits [24], supporting multi-component approaches that target both individual skills and the learning environment. However, given the cross-sectional design, we cannot establish temporal direction, and it remains possible that lower self-esteem is a consequence rather than a cause of suicidality and self-injury. While these findings highlight self-esteem as a promising correlate, they do not demonstrate that interventions to increase self-esteem will necessarily reduce suicidal behavior or self-injury. Longitudinal and interventional studies are needed to determine whether modifying self-esteem leads to durable reductions in suicide-related outcomes among medical students.

### 5.4. Health-Related Risk Factors: Smoking, Chronic Disease, and Mental Illness

The associations between smoking and both outcomes (AOR = 2.27–2.45) are consistent with literature documenting smoking as both a marker of psychological distress and a potential contributor to mood dysregulation through nicotine-related neurobiological mechanisms [40,41]. Given that 20.7% of our sample reported current smoking—a notable prevalence for a health professions population—tobacco cessation support integrated into student wellness programs may yield dual benefits for physical and mental health.

Mental illness diagnosis emerged as a strong predictor of both outcomes, with those diagnosed demonstrating approximately twice the odds of endorsement. This finding underscores the critical importance of accessible, destigmatized mental health services for medical students. The 18.2% of students reporting prior diagnosis represents a substantial population requiring ongoing support, yet help-seeking barriers including stigma, confidentiality concerns, and fear of academic or licensure repercussions remain formidable obstacles in medical education globally and may be amplified in cultural contexts emphasizing collectivism and family honor [18,42,43].

### 5.5. Living Arrangement and Social Isolation

Living alone emerged as a risk factor specifically for suicidal behavior endorsement, with 81% elevated odds compared to students living with family. Social isolation and lack of daily family contact may diminish access to informal emotional support and early intervention when distress emerges. In the Saudi context, where the majority of students traditionally live with extended family during university studies, those living alone may represent a particularly vulnerable subgroup warranting proactive outreach.

### 5.6. Divergence Between Suicidal Behavior and Self-Injury Predictors

An important observation from our analysis is the partial divergence in predictor profiles between suicidal behavior and self-injury endorsement. Pre-clinical training stage was independently associated with suicidal behavior (AOR = 1.69, *p* = 0.019) but not self-injury (AOR = 1.24, *p* = 0.350) after covariate adjustment. Conversely, younger age (18–20 years) was a significant predictor of self-injury (AOR = 2.12, *p* = 0.002) but not suicidal behavior. These patterns suggest that while the two outcomes share substantial overlap and common risk factors, they may possess distinct developmental and etiological features requiring differentiated prevention approaches.

The age-specific association with self-injury aligns with literature documenting NSSI as predominantly emerging during adolescence and young adulthood, with onset typically declining after the early twenties [44]. The youngest students in our sample may have initiated self-injury behaviors prior to medical school entry, with the construct capturing established patterns rather than behaviors emerging in response to medical training specifically.

### 5.7. Implications for Medical Education

While some Saudi medical colleges have implemented orientation programs for incoming students, these typically focus on academic logistics and curriculum overview rather than psychological preparedness and coping skills development. Structured transition support programs—including stress management training, study skills workshops, and proactive mentorship pairing during the first year—have shown promise in other medical education contexts [38] but are not yet systematically implemented across Saudi institutions.

Most Saudi medical colleges have some form of student counseling services, though the scope, staffing, and accessibility of these services vary considerably across institutions. Critically, utilization rates of available services are often low, driven by stigma, confidentiality concerns, and fear that seeking mental health care may negatively impact academic standing or future licensure [42].

Our findings carry several implications for medical education institutions in Saudi Arabia and comparable contexts. First, the identification of the pre-clinical period—and the second year specifically—as a window of heightened vulnerability argues for concentrated mental health resources and screening during this phase. Universal depression and distress screening upon matriculation and at annual intervals, with enhanced frequency during the second year, could enable early identification and intervention. Second, the protective effect of self-esteem suggests that wellness curricula emphasizing self-efficacy, resilience, and positive self-regard may confer preventive benefit. Peer mentorship programs connecting pre-clinical students with senior students and residents who have navigated similar challenges could provide both practical guidance and identity affirmation. Third, the associations between mental illness diagnosis, smoking, and chronic disease with both outcomes indicate that students with identified health conditions require enhanced support and monitoring. Integration of behavioral health services with student health clinics, normalization of help-seeking, and explicit institutional messaging regarding confidentiality protections may reduce barriers to care. Fourth, our finding that living alone constitutes a risk factor suggests that housing policies and social connection initiatives merit consideration. Formal peer support programs, structured social activities, and check-in systems for students living independently could mitigate isolation-related risk. Finally, the substantial prevalence observed across all training stages—never falling below 12%—indicates that while targeted intervention during peak-risk periods is warranted, ongoing attention to medical student mental health throughout training remains essential. Institutional culture shifts toward openness about psychological struggle, reduction in stigma, and prioritization of student wellbeing alongside academic achievement represent necessary systemic changes.

Cognitive interventions targeting maladaptive thought patterns—such as catastrophizing about academic failure—may be particularly beneficial. Gangemi et al. [45] demonstrated that specific cognitive techniques can effectively reduce the overestimation of threatening events in non-clinical populations, suggesting that such approaches could be adapted for medical students experiencing disproportionate academic distress.

## 6. Limitations

Several limitations should be considered. First, the cross-sectional design precludes causal inference about relationships between training stage, self-esteem, and outcomes; the observed decline across years may reflect true protective effects, survivorship, cohort differences, or all three, and only longitudinal cohorts can clarify this. Future longitudinal cohort studies following medical students from matriculation through graduation would clarify whether the observed decline in endorsement reflects true protective effects of clinical immersion, survivorship bias, or cohort differences.

Second, suicidal behavior and self-injury were measured with brief single items rather than validated scales for suicidal ideation and non-suicidal self-injury, limiting comparability and allowing variation in how respondents interpreted the items; there was no clinical verification and no data on intent, frequency, or recency, so severity cannot be determined. The single-item measure did not differentiate between suicidal ideation, suicidal planning, and suicide attempts, precluding analysis of the ideation-to-action continuum. Future primary studies should employ validated instruments such as the Columbia Suicide Severity Rating Scale (C-SSRS) and the Inventory of Statements About Self-Injury (ISAS) to enable more precise characterization and cross-study comparability.

Third, convenience sampling via social media and institutional channels yields a non-probability, self-selected sample with no calculable response rate, so representativeness of Saudi medical students overall is uncertain and selection bias (over- or under-participation of highly distressed students) is possible. Further research employing probability sampling with calculable response rates would strengthen the generalizability of these findings.

Fourth, all variables were self-reported, raising the likelihood of social desirability and under-reporting for stigmatized issues such as mental illness, smoking, and self-harm, which means prevalence estimates may be conservative.

Fifth, as a secondary analysis, the study lacked important variables (e.g., academic stress, coping, religiosity, family psychiatric history, prior help-seeking), These include type and severity of mental illness, prior or current engagement with mental health services (including inpatient admissions), psychotropic medication use, alcohol and illicit substance use, and family psychiatric history. The absence of these variables leaves residual confounding unaddressed and highlights the need for primary studies that measure these domains comprehensively.

## 7. Conclusions

This study extends the existing literature on medical student mental health in three key respects. First, it provides the first multicenter prevalence estimates for suicidal behavior and self-injury endorsement among Saudi medical students, addressing a significant gap in regional epidemiological data. Second, the year-by-year analysis reveals a previously undocumented vulnerability window during the second year of training—a finding with direct implications for the timing of preventive interventions. Third, the identification of high self-esteem as a robust protective factor across both outcomes highlights a potentially modifiable correlate that warrants interventional investigation. The identification of pre-clinical years as a period of heightened vulnerability, combined with the robust protective effect of self-esteem and modifiable risk factors including smoking and social isolation, provides an evidence base for targeted intervention development. Medical schools bear responsibility not only for producing competent physicians but for safeguarding the wellbeing of students during the demanding journey toward that goal. Our findings argue for concentrated mental health resources during early training, destigmatization of psychological struggle, and institutional commitment to student wellness as a foundational value. These findings complement the broader international evidence base while providing culturally specific data essential for informing mental health policy within Gulf medical education systems.

## Figures and Tables

**Table 1:** Sociodemographic and Clinical Characteristics of Participants (N = 1138).

Characteristic	n	%
Age group		
18–20 years	189	16.6
21–23 years	574	50.4
24–26 years	322	28.3
>26 years	53	4.7
Gender		
Male	506	44.5
Female	632	55.5
Academic year		
1st year	120	10.5
2nd year	133	11.7
3rd year	217	19.1
4th year	227	19.9
5th year	269	23.6
Intern	172	15.1
Training stage		
Pre-clinical (1st–3rd year)	470	41.3
Clinical/Intern (4th year–Intern)	668	58.7
Region		
Central	378	33.2
Northern	246	21.6
Southern	233	20.5
Eastern	142	12.5
Western	139	12.2
GPA		
4.75–5.00	238	20.9
4.50–4.74	238	20.9
4.00–4.49	324	28.5
3.00–3.99	288	25.3
<3.00	50	4.4
Living status		
With family	960	84.4
Alone	114	10.0
With colleague	64	5.6
Current smoker		
Yes	235	20.7
No	903	79.3
Chronic disease		
Yes	141	12.4
No	997	87.6
Diagnosed mental illness		
Yes	207	18.2
No	931	81.8
Self-esteem category		
Low (<15)	272	23.9
Normal (15–25)	769	67.6
High (>25)	97	8.5

**Table 2:** Overall Prevalence of Suicidal Behavior and Self-Injury Endorsement.

Outcome	n	%	95% CI
Self-injury endorsement	192	16.9	14.8–19.2
Suicidal behavior endorsement	193	17.0	14.9–19.3
Any endorsement	259	22.8	20.4–25.3
Co-occurring endorsement	126	11.1	9.4–13.0

Note. CI = confidence interval. Any endorsement = either or both outcomes. Co-occurring endorsement = both outcomes.

**Table 3:** Prevalence of Suicidal Behavior and Self-Injury Endorsement by Academic Year.

Academic Year	N	Self-Injury n (%)	Suicidal Behavior n (%)
1st year	120	26 (21.7)	23 (19.2)
2nd year	133	35 (26.3)	32 (24.1)
3rd year	217	37 (17.1)	46 (21.2)
4th year	227	33 (14.5)	37 (16.3)
5th year	269	33 (12.3)	33 (12.3)
Intern	172	28 (16.3)	22 (12.8)

Note. Chi-square tests: Self-injury χ^2^(5) = 17.94, *p* = 0.003; Suicidal behavior χ^2^(5) = 14.89, *p* = 0.011.

**Table 4:** Multivariable Logistic Regression: Factors Associated with Suicidal Behavior Endorsement.

Variable	AOR	95% CI	*p*
Training stage (pre-clinical vs. clinical)	1.693	1.090–2.631	0.019
Gender (female vs. male)	1.401	0.948–2.070	0.091
Age (18–20 vs. 21–23)	1.200	0.733–1.965	0.469
Age (24–26 vs. 21–23)	0.732	0.443–1.211	0.225
Age (>26 vs. 21–23)	1.331	0.598–2.962	0.484
Region (Northern vs. Central)	1.717	1.075–2.742	0.024
Region (Southern vs. Central)	1.191	0.720–1.971	0.497
Region (Eastern vs. Central)	0.922	0.499–1.703	0.795
Region (Western vs. Central)	1.273	0.725–2.237	0.400
Living alone (vs. with family)	1.808	1.077–3.037	0.025
Living with colleague (vs. with family)	1.819	0.967–3.420	0.063
Self-esteem: Normal (vs. low)	0.641	0.443–0.928	0.018
Self-esteem: High (vs. low)	0.192	0.066–0.560	0.003
Current smoker (yes vs. no)	2.270	1.514–3.404	<0.001
Chronic disease (yes vs. no)	2.192	1.406–3.419	0.001
Mental illness (yes vs. no)	2.353	1.566–3.534	<0.001

Note. AOR = adjusted odds ratio; CI = confidence interval; GPA = grade point average; AUC = area under the receiver operating characteristic curve. Model χ^2^(20) = 148.34, *p* < 0.001; AUC = 0.754; McFadden pseudo-R^2^ = 0.143. GPA categories not significant (all *p* > 0.05).

**Table 5:** Multivariable Logistic Regression: Factors Associated with Self-Injury Endorsement.

Variable	AOR	95% CI	*p*
Training stage (pre-clinical vs. clinical)	1.236	0.792–1.928	0.350
Gender (female vs. male)	1.230	0.842–1.798	0.285
Age (18–20 vs. 21–23)	2.124	1.316–3.427	0.002
Age (24–26 vs. 21–23)	0.912	0.561–1.483	0.711
Age (>26 vs. 21–23)	1.553	0.718–3.359	0.264
Region (Northern vs. Central)	1.463	0.919–2.328	0.109
Region (Southern vs. Central)	1.401	0.870–2.256	0.166
Region (Eastern vs. Central)	1.217	0.692–2.140	0.496
Region (Western vs. Central)	0.793	0.437–1.442	0.448
Living alone (vs. with family)	1.645	0.981–2.759	0.059
Living with colleague (vs. with family)	1.407	0.747–2.653	0.291
Self-esteem: Normal (vs. low)	0.801	0.551–1.165	0.246
Self-esteem: High (vs. low)	0.319	0.129–0.790	0.014
Current smoker (yes vs. no)	2.453	1.646–3.656	<0.001
Chronic disease (yes vs. no)	1.790	1.145–2.799	0.011
Mental illness (yes vs. no)	1.901	1.263–2.861	0.002

Note. AOR = adjusted odds ratio; CI = confidence interval; GPA = grade point average; AUC = area under the receiver operating characteristic curve. Model χ^2^(20) = 105.65, *p* < 0.001; AUC = 0.708; McFadden pseudo-R^2^ = 0.102. GPA categories not significant (all *p* > 0.05).

## Data Availability

The data are available on reasonable request. The anonymized dataset and analysis code used in this study can be obtained from the corresponding author upon reasonable request.
